# Signifying the Relationship Between Psychological Factors and Turnover Intension: The Mediating Role of Work-Related Stress and Moderating Role of Job Satisfaction

**DOI:** 10.3389/fpsyg.2022.847948

**Published:** 2022-05-03

**Authors:** Jinli Xue, Hao Wang, Meng Chen, Xiujuan Ding, Mengting Zhu

**Affiliations:** ^1^Research Center for Energy Economics, School of Business Administration, Henan Polytechnic University, Jiaozuo, China; ^2^Shandong Land and Space Ecological Restoration Center, Jinan, China; ^3^Natural Resources Bureau of Qingdao West Coast New Area, Qingdao, China; ^4^School of Management, Shanghai University, Shanghai, China

**Keywords:** emotional exhaustion, work engagement, role conflict, psychosocial risks, job satisfaction, turnover intentions

## Abstract

Human capital plays a significant role in an organization’s advancement. In recent years, emerging work-related psychological issues have become a critical factor, demanding considerable attention from management. As such, this study measures the role of job satisfaction in work-related stress and employees’ turnover intentions. There is a mediating relationship between work-related stress—such as emotional exhaustion, work engagement, role conflict, and psychosocial risks—and turnover intentions. The study used primary data collection techniques to gather data and purposive sampling to analyze the data. The study’s sample size consisted of 270 employees working in Chinese organizations. SmartPLS software was used to test the research hypothesis. The study results show the mediating role of work-related stress and moderating role of employees’ job satisfaction. Accordingly, the study provides implications for managers, encouraging them to take the necessary steps in controlling the turnover rate by enhancing employees’ morale (i.e., job satisfaction) and work engagement.

## Introduction

In recent years, psychological problems have emerged and gained prominence in the workplace, demanding effective human resource management (HRM). Today, a rise in psychological vulnerabilities has led to a reported increase in workers’ emotional instability (Reh et al., 2021). Emotional exhaustion refers to an individual’s ability to experience the overextended depletion of psychological resources. In particular, healthy labor requires a positive mindset for effective functioning. A heavy workload increases work-related stress while causing employees to face considerable health challenges (Jeon et al., 2018).

Workplace psychological issues (e.g., stress and depression) are strong predictors of high turnover. Many studies have uncovered that most ex-employees left their job due to having no ability to deal with work-related pressures. Consequently, this work-related stress has an effect on turnover intentions. Turnover intention alludes to an individual’s ability to withdraw from their work (Haque et al., 2019). Progressing work stress can be defined as excessive psychological demands and a drain in workforce energy. A high-stress level makes employees prone to experiencing psychological and emotional weariness, thus leading the individuals to depart from their job. In particular, an employee’s psychological state is an essential component driving the individual’s behavior and reactions. Attitude and behaviors are critical predictors of employees’ job satisfaction (Abdullah et al., 2021). As such, the previous research shows that emotional exhaustion ultimately affects job satisfaction, driving the employee’s intention to leave their employment (Lee and Chelladurai, 2018).

The workplace represents an opportunity for the development and growth among employees. When employees are confronted by quickly changing and more difficult working conditions, their ability to respond well is viewed as a key to job satisfaction (Bakker et al., 2003). Working environment stress is perceived as a far-reaching phenomenon that adversely influences employees’ wellbeing and prosperity and involves being overseen by employers. The relationship between poor work environment conditions and physical and mental manifestations has been found (Xanthopoulou et al., 2007).

In response to the identification of the individual, hierarchical, and cultural expenses of work environment stress, various initiatives and arrangements have been developed to counter it. The most common method is for employers to become accountable for checking employees’ degrees of openness to distressing emotional exhaustion, work engagement, role conflict, and psychosocial risks in regard to turnover intentions and its prevention. The normal working environment conveyed by employees is, for the most part, dependent on normalized self-report measures that are planned with a level of consensus (researching work factors that are generalizable to any work) to fit extremely heterogeneous work-related stress (Chang et al., 2009).

Work-related stress is a developing concern for the present work market because of the connection with work fulfillment, turnover (aim), work execution, and individual prosperity of laborers (Jain and Cooper, 2012). A few attributes of the advancement of work-related pressure in a medical setting include work stress, job satisfaction, high obligation, mental and actual responsibility, absence of occupation control, range of control and responsibility, absence of chances for scholarly and professional development, insufficient authority, insufficient social help by the administrator and additional partners, and poor medical attendant specialist cooperation.

Many studies have attempted to determine what conditions lead to the continuity of employees in a given workplace. The findings revealed that a level of occupation uncertainty ought to be considered. It gives a clearer picture of various work-related stress identities and their anticipated results (Mulki et al., 2008). By evaluating reactions to work uncertainty, the current review investigates how various elements of occupation weakness might be associated with workers’ job perspectives, mental prosperity, turnover intentions, and poor conduct.

Organizations are highly dependent on the workers’ mental prosperity. Human needs and interests (e.g., cognitive) significantly influence the individuals’ welfare (i.e., psychical and psychological) (Mohsin et al., 2021). As a result, it is vital to consider work-related issues (e.g., stress, turnover, and job dissatisfaction) to enhance the workers’ perspective toward their job role.

Undoubtedly, psychological instability causes several health problems, from decreasing employees’ productivity to increasing the dropout rate. Employee turnover is a significant predictor of high psychological threat (i.e., emotional exhaustion). As a critical contributor to employee turnover, emotional exhaustion must be well understood to foster employee job satisfaction. However, compared with the considerable information available on employees’ psychological wellbeing, there is little research on emotional exhaustion and employees’ turnover (Edmondson et al., 2019). Indeed, understanding and managing the labor force demand detailed research on employee psychological welfare and turnover rate.

Therefore, this study investigates the impact of emotional exhaustion, work engagement, role conflict, and psychosocial risks on turnover intentions. Secondly, it aims to measure the mediation of work-related stress on emotional exhaustion, work engagement, role conflict, psychosocial risks, and turnover intentions. Thirdly, it analyzes the effect of work-related stress on turnover intentions and measures the moderation of job satisfaction between them.

Indeed, this study is aimed to fill the research gap by presenting an essential practical guide for management, suggesting ways to take the necessary steps to mitigate excessive turnovers. This deficiency should be seen as an opportunity for future researchers (Ajaz et al., 2020) to contribute to employees’ wellbeing. Therefore, to establish effective management strategies, management should ensure the successful functioning of employees’ cognitive ability, influencing the firms’ growth (Shah et al., 2021). Consequently, the significance of this research topic enables future researchers to perform significant studies on emotional exhaustion in the workplace setting. Additionally, it assists market experts in managing human capital to guarantee high job satisfaction.

The study is outlined as follows: the first section briefly introduces the research topic; Section “Literature Review” (i.e., the literature review) provides a detailed view of previous studies. Along with this, the methodology section (i.e., section “Methodology”) explains the research tools adopted to perform the study analysis. Section “Data Analysis” illustrates the study results, while Section “Discussion” discusses the significant research findings. Lastly, Section “Conclusion” concludes the study by summarizing the critical aspects of the study findings.

## Literature Review

### Emotional Exhaustion and Turnover Intentions

The consumption of a worker’s energy and substantial mental pressure influence his/her mental condition and, taken together, can be considered emotional exhaustion. Yadav and Pathak (2016) characterized emotional exhaustion as when “laborers feel they are presently not ready to give themselves at a mental level.” The employees feel drained, exhausted, and their energies are depleted; these sentiments become ongoing and durable, and they are viewed as depleted in their ability.

Emotional exhaustion is common in the workplace (Hang-Yue et al., 2005). This leads to problems because emotional exhaustion causes the employees to be unable to take the expected interest in their work, and this can cause great difficulties if the issue becomes persistent. Bakker et al. (2005) assessed and identified aspects of burnout and reasoned that emotional exhaustion is a long-term mental illness, not simply fatigue, even though the symptoms can be the same.

Judge and Hurst (2008) applied one aspect of the Copenhagen Burnout Inventory, passionate fatigue, to evaluate the degree of burnout among workers with work-related stress. Additionally, Stumpp et al. (2009) embraced a one-layered methodology and utilized just the elements of passionate fatigue and emotional exhaustion to assess the level of burnout. A few outcomes from work stress include direct impacts, such as poor work execution. Studies have found emotional exhaustion to be essentially connected with a few business-related results, i.e., deliberate turnover among workers, poor work execution (Kim et al., 2015), and high turnover expectations (Judge et al., 2012).

Indeed, today, the concept of job turnover has become a profound concern of HRM in their attempts to ensure employee wellbeing and performance. Turnover is one of the biggest management problems that prevent individuals from performing their job (Haque et al., 2019). A high turnover is linked to several psychological problems, such as poor productivity, job dissatisfaction, emotional exhaustion, and depressive symptoms. Therefore, the research contributing to the literature of psychology states that emotional exhaustion acts as the central dimension of increased turnover intention (Caesens and Stinglhamber, 2019).

Accordingly, a significant number of works in the HR discipline have distinguished authoritative responsibility as the main forerunner of turnover aim and employee turnover intentions (Bowling et al., 2012). The idea of passionate depletion has been related to various work results; for example, work withdrawal, low responsibility, and the aim to leave. Primarily, employees engage in withdrawal cognition mainly to minimize their psychological burden. The study reveals that employees who experience high emotional exhaustion show high intention to leave the organization, thereby increasing concern for one’s mental wellbeing (Yuniasanti et al., 2019). Emotional exhaustion is the prominent factor that drives the employees’ intention to leave the organization. Further, emotional exhaustion prompts decreased viability and low usefulness in the workers who do stay with their employer. Indeed, increasing emotional exhaustion compels employees to adopt management strategies to fight against their escalating psychological vulnerabilities. Hence, the study suggests to establish a firm foundation to counter this problem, thus reducing the organizations’ burnout rate (Scanlan and Still, 2019). This review will recognize the relationship, as reflected by the following hypothesis:

H1: There is a significant relationship between emotional exhaustion and turnover intention.

### Work Engagement and Turnover Intentions

Work engagement, the optional connection of employees with their job, addresses the nature of connection as far as three aspects in particular: life, devotion and ingestion, and amount of connection (Kammeyer-Mueller et al., 2009). While researchers concur that connecting with workers has positive ramifications for the workers and also the business, the lack of connected workers has generated higher turnover intentions. Given the bleak circumstance regarding commitment and the capacity of connected employees to assist their workplace with accomplishing its objectives, it is necessary to investigate the elements that affect and impact work engagement, and as such, turnover indentions, in an organization.

An organization cannot gain success unless its employees exhibit a high commitment to achieve business objectives. Employees are the critical agents driving the organizations’ performance. Consequently, today’s employees must work hard to gain business progression. In this regard, employees’ commitment works best in connecting them to their organization. One study shows that high work commitment strongly determines the employees’ turnover intention (Shahid and Ahmed, 2020). Harris et al. (2009) expressed that expectation is identified with the employees’ work engagement. Their aim to leave their job relates to how well the organization is dealing with this aspect.

Barbuto and Bugenhagen (2006) have demonstrated that being connected with employees will generally have decreased the turnover rate. A few studies have measured employee work engagement against turnover intentions. Harris et al. (2009) took Kahn’s work as a beginning stage on this topic. Bakker and Demerouti (2007) suggested that work engagement addresses a state in which individuals bring in their selves during their job roles, contribute individual energy, and experience an enthusiastic association with their work. Similarly, Bowling et al. (2010) characterized work engagement as a positive perspective that employees have of their organization.

Yadav and Rangnekar (2016) found that high work engagement predicted an actual transfer to another company. In particular, the employees who feel emotionally connected to their work are less likely to leave their organization. In explaining this notion, the study shows that professional commitment (i.e., work engagement) significantly determines the employees’ intention to withdraw from their present employment (Chang et al., 2019). Work engagement infuses the feeling of excitement among individuals, making them feel excited about even the most challenging work task, thereby, influencing their intention of withdrawal.

In conclusion, numerous studies have found a significant link between work engagement and intention to leave employment. Hence, this relationship has contemporary importance by demonstrating that work engagement boosts employees’ morale, thereby, leading them to display greater work dedication and conviction. Thus, it is stated that:

H2: Work engagement is positively related to turnover intentions.

### Role Conflict and Turnover Intentions

Hang-Yue et al. (2005) state that turnover has been estimated as the most real and broad issue that numerous employers face among their employees. Johnson et al. (2008) establish that there ought to be three aspects in job role conflict. Role conflict refers to a clash with respect to conduct required by the job obligations and responsibilities to family and work. It is clarified as the time needed to execute both requests (family or work) and is shown by the time allocated for family and for work. Role conflict is a crucial factor compelling employees to resign from their job. It alludes to individuals’ unpleasant job experiences that involve a clash between social and professional status (Anand and Vohra, 2020). These role conflicts exert immense mental stress on individuals, thus adversely influencing their intention to stay connected with the company (Dai et al., 2019). Likewise, Swann et al. (2003) review uncovers that role conflicts are viewed as emphatically identified with work-related stress and adversely identified with work fulfillment, work association, pay, management, and working conditions that increase turnover intentions. In their example of teachers, they state that this explains the turnover rate in that profession according to the organization’s point of view.

The attributes of turnover intentions are authoritative ineffectualness, deficient assets, and disappointment. Employees may be leaving the workforce permanently or will often move to another employee with better requirements. For both, turnover intention arises due to work pressure according to the degree to which the work obligations cannot be satisfied. Conversely, it is countered by employees’ plans to remain in their occupation through fulfillment and responsibility, which undermine turnover intentions (Turnipseed and Bacon, 2009).

Employee management must consider poor working conditions and the conflicts that arise and worker-based elements, such as occupation fulfillment, individual conviction, and development. For example, external factors, such as payment levels in comparable positions, the status and regard given to workers, impact the dropout of educators in private auxiliary schools. Harris et al. (2009) state that most work-related stress arises from contention among employees that contradicts their own role conflicts; lack of resources for the appropriate execution of job tasks; deficient skills to fulfill their job needs; insufficient independence to make choices; and a sensation of underutilization.

Lewin and Sager (2007) clarified the impacts of stressors, such as job conflict, job struggle, work-over-burden, work-family struggle, work pressure, work fulfillment, authoritative responsibility, and worker turnover expectation and intentions. The review uncovered that turnover aim consistently impacts role conflict and turnover intentions. On the other hand, role conduct and occupation stress also correspond to the turnover intentions.

Undoubtedly, employee turnover is an ongoing trend whereby individuals withdraw from their jobs. In the current phase of employment, employees face several psychological challenges that can produce distressing outcomes. Despite offering numerous employee incentives, job conflict (e.g., between social and professional lives) can make employees quit their employment. Due to the increasing work-life imbalance, Mansour and Tremblay’s (2018) study draws our attention toward increased employee turnover intention. The research states that employees tend to quit their job due to progressing personal and social conflicts. Accordingly, various scholars reveal that excessive workload with progressing work commitments increases workplace stressors, thus boosting employees’ intention to leave the organization (Awan et al., 2021). Therefore, it is found that:

H3: There is a significant relationship between role conflict and turnover intentions.

### Psychosocial Risks and Turnover Intentions

Turnover intentions are characterized as a worker’s tendency to leave their workplace. Turnover has been viewed as possibly the consequence of an intention to quit (van Doorn and Hülsheger, 2015). Worker devotion and expectations to remain with the employer are elements of managers’ psychosocial behavior that risk employer stability. The basic rationale of a connection between psychosocial risks and turnover aims, as indicated by Kammeyer-Mueller et al. (2009), is that people tend to pull out from upsetting circumstances.

Employees are not interested in working in an environment that does not ensure mental wellness. Psychological risk leads to a high burnout rate. The literature reveals that the psychological consequences enable the workplace characteristics to be a critical source of mental imbalances, thus making the individual unable to cope with the workplace’s requirements. Managers demand that employees bear the workplace pressure by handling a high workload, thereby, causing decreased individual wellbeing (Pace et al., 2021) and higher turnover intention (Knani et al., 2018). In particular, the psychological risk associated with an excessive workload causes extreme stress to individuals, subsequently influencing their work activity. Employees’ exposure to a psychological threat concerning their work requirement makes individuals depart from their job. As such, the research suggests that psychological challenges linked to work engagement pose a high health risk, increasing the dropout rate (Soto-Rubio et al., 2020).

Therefore, work-related risks, through any stressor, will incite withdrawal reactions. States of occupation instability might have negative ramifications for workers’ work execution. Some studies likewise uphold a relationship between psychosocial risks and turnover intentions. Past experimental work proposes a positive affiliation; the connection between work psychosocial risks and hazardous conduct is not shown to be immediate (Vigoda-Gadot and Kapun, 2005).

This can clarify the critical relationship between the psychosocial risks and turnover intentions. Workers with specific experience and higher capabilities may desire to look for various promotional openings. Managers with higher status may be relied upon to have a steadier work circumstance. This can emphatically impact their goal to stay in their workplace (McNatt and Judge, 2008). Psychosocial risks for job burnout include job struggles, work/time pressure, lack of choice power, poor work arrangements, and work-home impedance. Psychosocial risks for turnover expectations were seen as job significance, lack of choice power, work arrangements, staff assets, work/home impedance, and professional stability. Work/time pressure was seen as influential, as were work/home impedance and staff assets. These psychosocial risk factors were related to work pressure and prosperity. Further, the literature review results support the idea that workers face clashes about parts of their work role, and this causes stress and greater turnover intention. As such, it is found that:

H4: Psychosocial risks are positively related to turnover intentions.

### Meditating Role of Work-Related Stress

Stress is a far-reaching and expensive issue in present-day society, especially in the work environment. Past studies propose that stress has a relationship with turnover intention. This conceptualization concerns the earlier agreed idea that expanding pressure levels in the present place of employment might prompt a choice to leave the poor workplace environment, as it creates work-related stress. Stress can bring about non-attendance, high staff turnover, poor attitude, and a potential increase in accidents because of human mistakes. It is comprised of occupation requests, work control, administrative help, peer support, brutality relationship, job clearness, and change.

Further, ongoing studies on pressure have highlighted the significant role of a person’s intellectual capacity (pressure, adapting style) and mental qualities (for example, confidence, idealism, and neuroticism) in handling pressure (Bakker et al., 2005). Work-related stress is a mental idea that has received a lot of consideration in the last 20 years, as stress rises and employers witness their employees’ apparent capacity to adapt to these stresses. The literature reveals that pressure can prompt undesirable results in terms of workers’ physical wellbeing, behavior, and position within a company. Work-related stress is a significant reason behind work-related chronic sickness, helplessness, and human errors due to emotional exhaustion. Emotional exhaustion is the most critical component that escalates the dropout process. In particular, emotional exhaustion adds to the feeling of occupational stress. Prolonged stress influences an individual’s turnover intention. Enduring stress factors lead to a rise in absenteeism, psychological distress, and emotional exhaustion. In support of this, the literature shows that increased stress symptoms (i.e., psychological risk) have devastating consequences for individuals’ psychological health, thus increasing the likelihood of them for quitting the job (Knani et al., 2018).

Mulki et al. (2008), utilizing two distinct examples, tried to make explicit connections between work stressors and result factors. The exploration model proposes four focal areas of the work circumstance: work content, working conditions, social and work relations, and business states with employee conflict. The role conflict exploration model outlines three significant result factors: natural work inspiration, enthusiastic depletion, and increased conflicts between employees. The outcomes largely upheld the proposed notion of connections and were invariant across two examples. Additionally, Mulki et al. (2008) researched the connections between stress, social help, and expectations. Workers with more significant pressure levels were bound to consider leaving, while those receiving more prominent social help were more forthright. Social help did not stop the impacts of hierarchical pressure. However, it had some impact in buffering the impacts of work-family struggle.

Work-related stress immensely influences individuals’ psychological wellbeing, making it difficult for employees to manage their professional and personal lives. Hence, the literature states that job conflict aggravates stress symptoms, ultimately increasing the turnover rate (Soelton et al., 2020). In an organization, work demands the individuals to perform their tasks with a healthy mindset (i.e., emotional stability). Inadequate health caused by limited personal resources (e.g., efforts and time) affects the psychological state of workers (Fonseca et al., 2021), thereby leading the individual to look to changing jobs.

Work-related stress has turned into an inexorably significant general medical problem, affecting physiological and psychological wellbeing. Business-related pressure has been demonstrated to be profoundly connected with numerous indications of depression, such as sleeping disorders, diminished focus, exhaustion, lack of energy, and feelings of uselessness (Chang et al., 2009).

Work-related stress pressure is profoundly connected with an expanded danger of specific mental issues, such as poor work engagement (Johnson et al., 2008). Earlier reviews exploring the relationship between work-related stress and discouragement showed that this relationship is intricate. Future investigations should consider the systems that add to the increased likelihood of mental problems among workers with significant degrees of work-related stress. As such, the following hypotheses are proposed:

H5: A significant relationship exists between work-related stress and turnover intentions.
H6: Work-related stress mediates between emotional exhaustion and turnover intentions.
H7: Work-related stress mediates between role conflict and turnover intentions.
H8: Work-related stress mediates between psychosocial risks and turnover intentions.
H9: Work-related stress mediates between work engagement and turnover intentions.

### Moderating Role of Job Satisfaction

Many studies depict the important role of job satisfaction as a moderator (Swann et al., 2003). Job satisfaction is a vital variable that has a unique implication when considering the effects of various antecedents on work-related stress and turnover intentions, and it is moderated through job satisfaction. Lambert et al. (2007) determined that work/job satisfaction considerably affects turnover intentions as a moderator. The latest research has confirmed that job satisfaction moderates the relationship between work-related stress and turnover intentions (Harris et al., 2009). Bolstering this view, the current study has indicated that work satisfaction plays a central role in moderating the relationship between work-related stress and turnover intentions. The study found that work satisfaction moderates the relationship between employees’ work-related stress and turnover intentions to some extent.

Therefore, the study shows that work stress decreases an employee’s job contentment, thereby, increasing the individual’s intention to withdraw from their present job (Edmondson et al., 2019). This affirms a previous study conducted on the way that work-related stress and job satisfaction moderate the relationship between them (Harris et al., 2009). Therefore, it is necessary to minimize the psychological risk associated with job satisfaction. Work satisfaction ensures the fulfillment of employees’ needs in the workplace. Hence, to protect against these progressing psychological threats, job stress needs to be controlled to achieve high employee satisfaction. This leads to the following hypothesis:

H10: Moderation of job satisfaction exists between work-related stress and turnover intentions.

**FIGURE 1:**
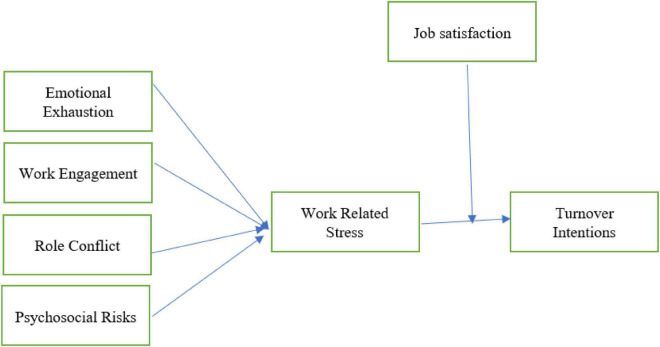
Conceptual framework.

Lewin and Sager’s (2007) study model addresses job over-burden, hierarchical limitations, and job struggles. Openness to these elements showed consequences for emotional exhaustion, work engagement, role conflict, and psychosocial risks on turnover intentions. Figure 1 illustrates the study’s conceptual framework.

## Methodology

This study has followed a quantitative approach and conducted a survey for data collection. It used numerical values through questionnaire surveys and analyzed them through SPSS and SmartPLS. The data were collected from employees who were working in Chinese organizations. The researcher used the purposive sampling technique to collect data. The study disseminated 300 questionnaires; of those, 270 were received for research. A sample of 50 respondents led the pilot study to check the reliability of the items. Further, the data have been processed through SPSS and Smart PLS, which were used to measure the suggested hypotheses with relation to emotional attachment theory.

The total number of respondents was 300; 120 (44%) were women and 150 (56%) were men. In terms of the education of the respondents, 27 have diplomas (10%); 105 (38.88%) had bachelor’s degrees, 79 (29.259%) had master’s degrees, 49 (18.14%) had MPhil, and 10 (3.703%) were with a doctorate Ph.D.

The scale for the construction of work-related stress was adopted from the study of Lambert et al. (2007), containing three items. It measured the effect of emotional exhaustion, work engagement, psychosocial risks, and role conflict in the current study. The moderation of job satisfaction consisted of three items, and it was adapted from the study of Smith et al. (1969). The turnover intentions consisted of four items, which were taken from the study of Jacobs and Roodt (2008). The scale for the role conflict was taken from the study of Rizzo et al. (1970), and it comprises three items. The scale for the psychosocial risks was taken from the study of Jacukowicz and Wezyk (2018), and it has four items.

The scale for emotional exhaustion was taken from Brenninkmeijer’s (2003) study, and it has three items. The scale for work engagement was taken from the study of Prins et al. (2009), and it has three items. The researcher tested the variables’ validity and reliability to ensure any ambiguities in the questionnaire were avoided.

## Data Analysis

This research study has used the structural equation modeling (SEM) approach in the SmartPLS 3.3 latest software version. SEM is used to measure the relationship between variables. The study measures the coefficient values and the relationship between them. The researcher has used a cross-sectional study to measure the relationship. The construct has three independent variables: a mediator and a dependent variable. The SEM consists of two steps of analysis of the partial least squares (PLS) algorithms, Bootstrapping, PLS logarithms are the weighted vector-based regression analysis models, which show coefficient values.

### Measurement Model Assessment

This study used SmartPLS software to analyze the measurement model that shows the Cronbach alpha, composite reliability, factor loading, and average extracted values. The reliability between constructs measured by the Cronbach alpha values should be between 0.6 and 0.90. Another method is the average variance extracted (AVE) that should be greater than 0.5; if any value is less than 0.5, it considered some items deleted and changes.

According to Fornell and Larcker (1981), cross-loadings and heterotrait-monotrait ratio (HTMT) and factor loading occur when one factor depends on more than other factors. It reflects the dependency of the data. HTMT ratios show the correlation among variables; its range is − 1 to + 1. It should be less than one, and it considers a strong relationship between two variables at a significance level of 0.01. Furthermore, this study has also measured effect size F and R-square, which shows the data’s significance and dependency. Table 1 shows Fornell-Larcker criterion values.

**TABLE 1:** Fornell-Larcker criterion.

	EEX	WEG	ROC	PSR	WRS	JOS	TOI
Emotional exhaustion	0.543						
Work engagement	0.512	0.526					
Role conflict	0.416	0.582	0.628				
Psychosocial risks	0.712	0.612	0.518	0.612			
Work related stress	0.417	0.732	0.710	0.619	0.731		
Job satisfaction	0.616	0.715	0.601	0.505	0.711	0.572	
Turnover intentions	0.623	0.619	0.711	0.694	0.605	0.610	0.614

The association of emotional exhaustion with turnover intentions is strong, as shown by the result 0.623 and confirmed the acceptable association between constructs according to the criteria in this study. Further, the result 0.619 showed a strong and significant association of safety of work engagement with turnover intentions greater than 0.5. The value 0.711 indicated that the relationship of role conflict with turnover intentions is significant and strong, and the value 0.694 showed that psychosocial risks are strongly associated with turnover intentions. Therefore, work stress has a strong association with turnover intentions established by the result 0.605, and job satisfaction has a strong relation with turnover intentions that is confirmed by the result of 0.610 (Table 1).

Table 2 indicates the HTMT values of variables; it has criteria comparing the threshold that should be less than 0.85. The results showed that emotional exhaustion has a strong association with turnover intentions. Moreover, the results indicated a strong association between work engagement and turnover intentions. The study showed a significant relationship between role conflicts and turnover intentions that meet the predefined criteria. Psychosocial risks have strong relation with turnover intentions with a value of 0.519. The value of 0.611 confirmed the strong relationship between work-related stress and turnover intentions. Further, the results showed a strong relationship between job satisfaction and turnover intentions.

**TABLE 2:** Heterotrait-monotrait (HTMT).

	EEX	WEG	ROC	PSR	WRS	JOS	TOI
Emotional exhaustion							
Work engagement	0.512						
Role conflict	0.517	0.635					
Psychosocial risks	0.589	0.690	0.457				
Work related stress	0.562	0.465	0.562	0.615			
Job satisfaction	0.701	0.513	0.718	0.506	0.617		
Turnover intentions	0.581	0.599	0.612	0.519	0.611	0.629	

The composite reliability (CR) values should be greater than 0.7, which shows the reliability and consistency of the data. AVE should be greater than 0.5, and CR value should be greater than 0.5 of a variable. It shows a high significance level and ensures the threshold level of the study. Table 3 shows study variables’ reliability and validity.

**TABLE 3:** Reliability and validity analysis.

	Items	Loadings	Cronbach’s alpha	rho_A	CR	AVE
Work engagement	WEG1	0.781	0.715	0.922	0.709	0.687
	WEG2	0.812				
	WEG3	0.762				
Emotional exhaustion	EEX1	0.716	0.823	0.882	0.729	0.713
	EEX2	0.709				
	EEX3	0.720				
Psychosocial risks	PSR1	0.784	0.823	0.873	0.762	0.763
	PSR2	0.619				
	PSR3	0.801				
	PSR4	0.811				
Role conflict	ROC1	0.710	0.823	0.722	0.772	0.817
	ROC2	0.768				
	ROC3	0.815				
Turnover intentions	TOI1	0.789	0.763	0.865	0.872	0.785
	TOI2	0.707				
	TOI3	0.891				
	TOI4	0.687				
Job satisfaction	JOS1	0.791	0.783	0.781	0.719	0.618
	JOS2	0.784				
	JOS3	0.786				
Work-related stress	WRS1	0.835	0.809	0.773	0.732	0.679
	WRS2	0.825				
	WRS3	0.882				

### Structural Model

Table 4 demonstrates the relationship of emotional exhaustion and work-related stress (*t*-value = 4.067, *p* = 0.001). The study hypothesis is accepted because work engagement has a significant positive relationship with work-related stress (*t* = 3.782, *p* = 0.000). There is a positive and significant relationship between role conflict and work-related stress (*t* = 2.445, *p* = 0.001). Similarly, psychosocial risks are positively related to work stress (*t* = 3.781, *p* = 0.000).

**TABLE 4:** Hypothesis results.

Hypothesis	Std Beta	*SD*	*T*-values	*P*-values	ULCI	LLCI
Emotional exhaustion— > Work related stress	0.234	0.204	4.067	0.001	0.001	0.123
Work engagement— > Work related stress	0.568	0.215	3.782	0.000	0.10	0.172
Role conflict— > Work related stress	0.321	0.045	2.445	0.001	0.043	0.213
Psychosocial risks— > Work related stress	0.567	0.092	3.781	0.000	0.113	0.165
Emotional exhaustion— > Turnover intentions	0.432	0.093	3.265	0.002	–0.013	0.021
Work engagement— > Turnover intentions	0.232	0.283	4.171	0.001	–0.013	0.121
Role conflict— > Turnover intentions	0.268	0.230	5.731	0.000	0.10	0.172
Psychosocial risks— > Turnover intentions	0.532	0.403	13.265	0.003	–0.113	0.221
Emotional exhaustion— > Work related stress— > Turnover intentions	0.291	0.235	5.813	0.001	0.103	0.189
Work engagement— > Work related stress— > Turnover intentions	0.235	0.367	2.882	0.001	0.121	0.216
Role conflict— > Work related stress— > Turnover intentions	0.172	0.283	3.655	0.001	0.014	0.311
Psychosocial risks— > Work related stress turnover intentions	0.345	0.412	2.732	0.000	0.001	0.210
Work related stress— > Turnover intentions	0.526	0.324	9.421	0.000	0.003	0.195
Work related stress— > Job satisfaction— > Turnover intentions	0.354	0.340	3.813	0.001	–0.021	0.128

Table 4 also represents the impact of emotional exhaustion on turnover intentions (*t*-value = 3.265, *p* = 0.000). Work engagement has a significant positive relationship with work-related stress (*t* = 4.171, *p* = 0.001). The relationship between role conflict and work-related stress has a positive and significant relationship (*t* = 5.731, *p* = 0.001). Psychosocial risks are positively and significantly related to work stress (*t* = 13.265, *p* = 0.000).

**FIGURE 2:**
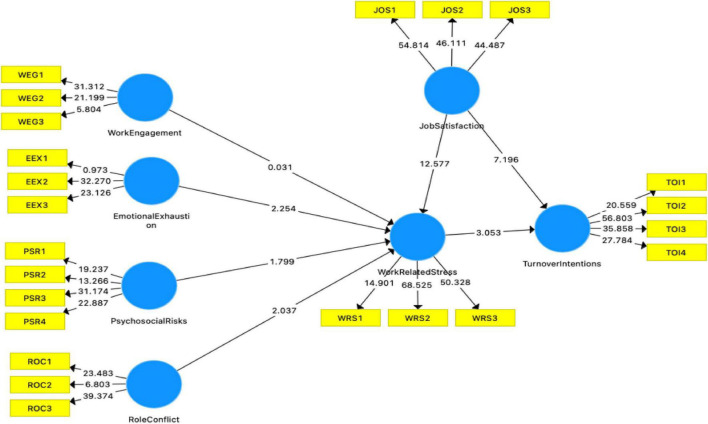
Structural equation model.

Work-related stress has a significant relationship between emotional exhaustion and turnover intentions (*t* = 5.813, *p* = 0.001). The mediation of work-related stress has a significant relationship between work engagement and turnover intentions (*t* = 2.882, *p* = 0.001). The mediation of work-related stress has a significant relationship between role conflict and turnover intentions (*t* = 3.655, *p* = 0.001). The mediation of work-related stress has a significant relationship between psychosocial risks and turnover intentions (*t* = 2.732, *p* = 0.000). In addition, work-related stress has a significant relationship with turnover intentions (*t* = 9.421, *p* = 0.000). Job satisfaction moderates between work-related stress and turnover intentions (*t* = 3.813, *p* = 0.001). Figure 2 is a graphical representation of the structural model.

## Discussion

Significantly, in recent years, the notion of turnover intention has gained massive popularity in the various domains of management. Consequently, this growing attention has encouraged managers to understand and incorporate this concept for managing workplace problems (Li et al., 2019). In particular, this inherit section discusses the study outcomes in the light of the previous research reviews. Accordingly, for improving the understanding of increasing burnout rate, this current study shows concerns toward exploring the effectiveness of emotional exhaustion, work engagement, job conflict, psychological risk (i.e., stress), and job satisfaction on turnover status in Chinese organizations.

Emotional exhaustion refers to the decrease in an individual’s energy level, thus detrimentally influencing individuals’ psychical and psychological wellbeing. This cognitive strain makes individuals exhibit negative attitudes and behaviors while compelling the employees to quit the job (Caesens and Stinglhamber, 2019). In contrast, the study shows that employees who feel connected with their job invest their time and energy in work, substantially minimizing the likelihood of their withdrawal (e.g., absenteeism) (Park and Johnson, 2019). Hence, the prior studies support our research findings that record a significant relationship between emotional exhaustion and work engagement to turnover intention. Based on these, studies we have fundamentally accepted the hypotheses H1 and H2.

Furthermore, job conflict is a critical component escalating the turnover. In explaining this notion, the study highlights the reality that the psychological imbalance experienced by the employees (i.e., personal and professional lives) makes the individual depart from the present employment (Zhang et al., 2019). The psychological factors make the work difficult causing numerous health challenges (Bergh et al., 2018), lower work commitment (Watanabe and Yamauchi, 2019), high dropout rate (Elshaer et al., 2018), and increased work-related stress (Junne et al., 2018). Perhaps, our study findings considerably support the prior studies while accepting all the hypotheses (e.g., H3, H4, and H5).

The study contributed to work-related stress and moderated by job satisfaction by measuring the impact of emotional exhaustion, turnover intentions, role conflict, and psychosocial risks on turnover intentions. When employees consider that they are just following orders from their superiors rather than their personal action, they experience a lack in the satisfaction level due to little independence and freedom. Self-determination exists when the workforce has some control over what they do and can decide how much effort they put into their work (Spector, 1986). Based on the regression model, meaning and impact are the most critical cognitions that contributed to the turnover intentions with work-related stress.

The study has found a significant and positive relationship as *t*-values are greater than 1.96, and all hypotheses are supported. The study found a significant mediating relationship of work-related stress between emotional exhaustion, turnover intentions, role conflict, and psychosocial risks on turnover intentions. Moderation effect of job satisfaction exists between work-related stress and turnover intentions, and *t*-value is greater than 1.96, and values of *p* are significant.

## Conclusion

Over the years, employee turnover has become a prime concern for HRM. Several factors play a critical role in accelerating the employees’ dropout rate. Psychological risk, job conflict, and high work stressors have massively disrupted the employees’ psychological wellbeing, substantially decreasing the employees’ satisfaction. Stress has been a critical element in regulating the turnover rate. The increasing job strain poses massive psychological problems (e.g., emotional exhaustion and distress), thus disengaging the employees from their work. Employees respond positively when their efforts, abilities, skills are well-acknowledged and appreciated by the management. In particular, employee satisfaction is necessary for building the employees’ connection with the job.

Based on the outcome analysis of this study, it can be stated that there is a moderating effect of job satisfaction in the relationship between work-related stress and turnover intentions. The mediation of work-related stress found that emotional exhaustion, turnover intentions, role conflict, and psychosocial risks have a significant relationship and effect on turnover intentions. The findings show the study’s direct and indirect relationships among variables and meditation of work-related stress.

In addition to that, the correlation between turnover intentions and work-related stress and moderated by job satisfaction is positive. The four cognitions of employees’ work-related stress have a significant positive relationship between turnover intentions. The relationship between emotional exhaustion, work engagement, role conflict, and psychosocial risks on turnover intentions and job satisfaction is significant and positive.

Therefore, after conducting a detailed review of the previous studies, the study concludes a significant relationship between emotional exhaustion, job conflict, psychological risk, and work-related stress with turnover intention. Indeed, the study findings are significantly predictable, influencing the turnover intention of Chinese employees. The study suggests that managers should focus on increasing the work commitment among the employees. It states that employees should be motivated to work in an interactive environment, thus enhancing individual satisfaction.

## Data Availability Statement

The raw data supporting the conclusions of this article will be made available by the authors, without undue reservation.

## Ethics Statement

Ethical review and approval was not required for the study on human participants in accordance with the local legislation and institutional requirements. The patients/participants provided their written informed consent to participate in this study.

## Author Contributions

All authors listed have made a substantial, direct, and intellectual contribution to the work, and approved it for publication.

## Conflict of Interest

The authors declare that the research was conducted in the absence of any commercial or financial relationships that could be construed as a potential conflict of interest.

## Publisher’s Note

All claims expressed in this article are solely those of the authors and do not necessarily represent those of their affiliated organizations, or those of the publisher, the editors and the reviewers. Any product that may be evaluated in this article, or claim that may be made by its manufacturer, is not guaranteed or endorsed by the publisher.
